# Supplementary material for: Proof-of-Concept Pilot Study on Comprehensive Spatiotemporal Intra-Patient Heterogeneity for Colorectal Cancer With Liver Metastasis
Source: Front Oncol. 2022 Mar 23;12:855463. doi: 10.3389/fonc.2022.855463 (PMC8986149; doi:10.3389/fonc.2022.855463)
Supplement: Supplementary file 1 [file Presentation_1.pptx]

## Slide 1
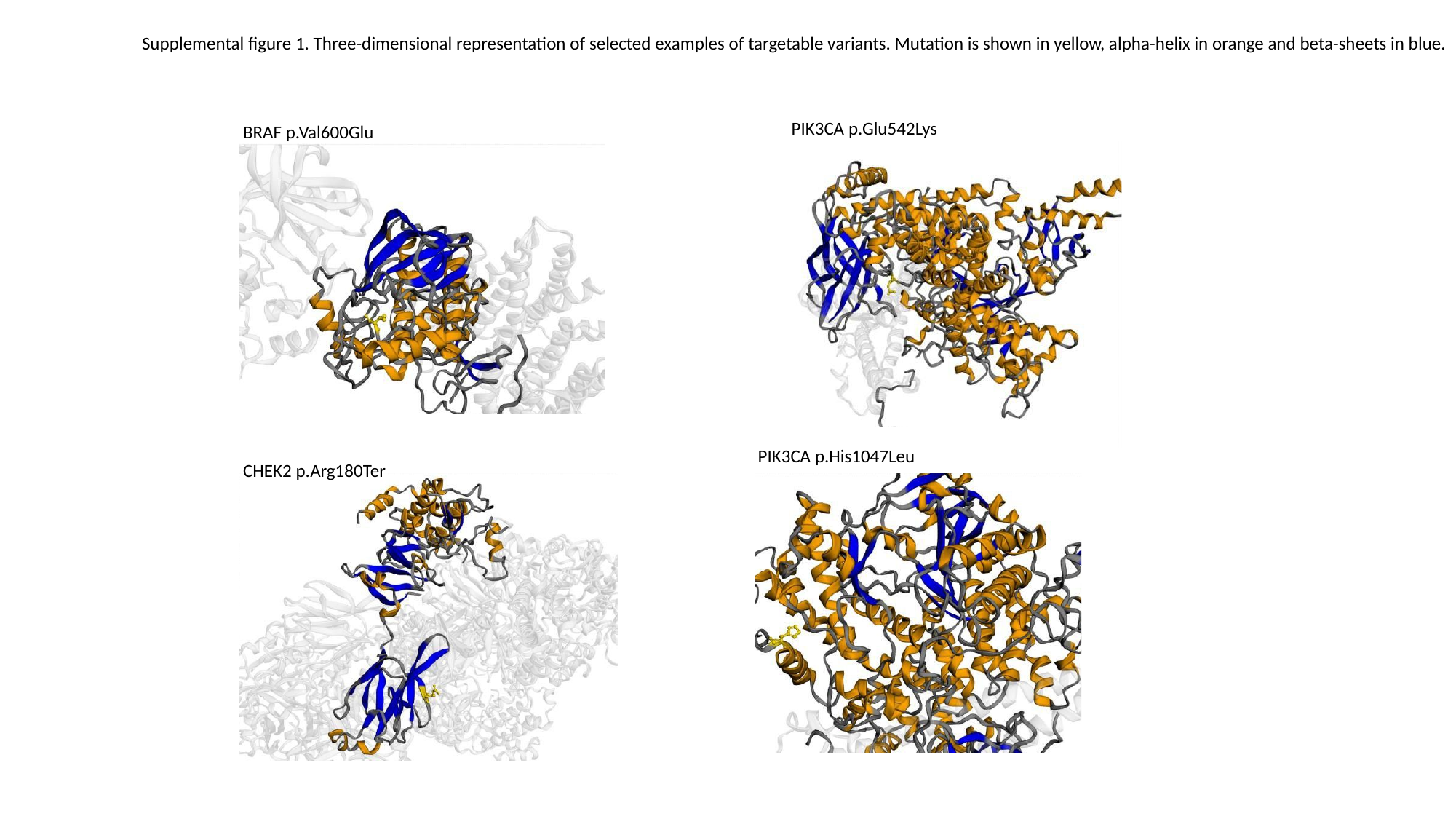

Supplemental figure 1. Three-dimensional representation of selected examples of targetable variants. Mutation is shown in yellow, alpha-helix in orange and beta-sheets in blue.
PIK3CA p.Glu542Lys
BRAF p.Val600Glu
PIK3CA p.His1047Leu
CHEK2 p.Arg180Ter
